# From planning to savings: CRC screening organization and cost-utility analysis of early CRC detection in Bulgaria

**DOI:** 10.1186/s13690-025-01810-1

**Published:** 2025-12-23

**Authors:** Hristo Krastev, Evgeni Mekov, Veneta Todorova, Georgi Slavchev, Adriana Dacheva, Slaveyko Djambazov

**Affiliations:** 1 HTA Ltd., Sofia, Bulgaria; 2 Medical University-Sofia, Sofia, Bulgaria

**Keywords:** CRC screening program, Bulgaria, Cost-utility analysis, Communication strategy, Organizational strategy, ICER

## Abstract

**Background:**

Colorectal cancer (CRC) is the most commonly diagnosed cancer in Bulgaria and the second leading cause of cancer-related death. Bulgaria has increased CRC mortality, highlighting the urgent need for effective screening interventions. This study evaluated the operational outcomes and cost-utility of Bulgaria’s first national CRC screening program.

**Methods:**

The screening program targeted individuals aged 50–74 years via the fecal immunochemical test (FIT). Free FIT kits were distributed primarily through laboratories and pharmacies, with placement guided by merchandising best practices to maximize visibility and uptake. A multichannel communication strategy, led by television, encouraged participation. Anonymous demographic, testing, and communication data were analysed to evaluate campaign performance. A centrally coordinated model, organized by the Lachezar Tsotsorkov Foundation, ensured consistency, efficiency, and national reach.

Cost-utility analysis (CUA) based on a Markov model with a lifetime horizon was conducted from the payer’s perspective, comparing screening versus no screening. Transition probabilities were sourced from published analyses in the Netherlands, the SEER database, and a Danish study. Probabilistic sensitivity analysis was used to assess the impact of parameter uncertainty.

**Results:**

A total of 93,381 individuals were screened, exceeding the original target by 86%. Of these, 14.2% tested positive. While women represented 63.3% of the participants, men had a higher positivity rate (18.2% vs. 11.9%). In the target population (individuals aged 50–74 years), 9,380 (14.4%) tests were positive.

The most influential communication channel was television (45.1%), followed by personal networks (24.6%).

The cost per participant was calculated at €7.84 (BGN 15.34), which includes the cost of the tests, management expenses and promotion of the screening program.

The results of the CUA estimates €67.07 (BGN 131.18) savings and +1.58 QALY gained per patient detected, projecting an incremental national gross domestic product contribution of up to €15.9 million (assuming full return to work capacity per quality-adjusted life year and actual productivity gains).

**Conclusion:**

A centrally coordinated, data-responsive, and well-communicated screening program can achieve high participation and early detection. The presented model offers a scalable cost-effective (feasible and economically justified) framework for national CRC prevention efforts in Bulgaria and other settings facing rising CRC burdens.

**Trial registration:**

Not applicable.

**Table:** 

Text box 1. Contributions to the literature
• This study provides the first evaluation of Bulgaria’s national colorectal cancer screening program via FITs.
• A nonprofit-led, centrally coordinated model can organize and manage screening more effectively than traditional public health structures can.
• This study demonstrates that merchandising-based kit placement and television-led communication substantially increased participation.
• Confirms the cost-effectiveness of CRC screening (€67 savings and +1.58 QALY per detected patient) and its projected positive economic impact.

## Background

Colorectal cancer (CRC) represents a major public health concern globally, as it is one of the most common types of cancer worldwide and is characterized by significantly high mortality rates [1, 2]. In Bulgaria CRC is the most frequently diagnosed malignancy and among the leading causes of cancer-related mortality [3]. According to GLOBOCAN data, CRC accounted for 15.5% of all newly diagnosed cancer cases in Bulgaria in 2022, making it the number one cancer by incidence and the second by mortality, with 2,759 deaths recorded that year [3]. Bulgaria is the only country in the European Union (EU) that has reported an increase in cancer-related mortality over the last decade—8% among men and 5% among women—while the EU average has experienced decreases of 10% and 5%, respectively [3].

CRC usually begins as noncancerous polyps in the colon or rectum, which are collectively referred to as CRC due to their similarities [4]. CRC is largely asymptomatic in its early stages, which significantly hinders timely diagnosis [5]. However, early detection significantly improves outcomes, with stage I five-year survival rates of approximately 90%, compared with 10–14% for stage IV patients [6]. Early detection is therefore crucial not only for improving patient outcomes but also for reducing the economic burden on healthcare systems. Compared with advanced disease, which requires chemotherapy, radiotherapy, and/or immunotherapy, treatment at early stages typically involves surgery with fewer complications, shorter recovery periods, and lower costs [6].

Screening methods such as the faecal immunochemical test (FIT), faecal occult blood test (FOBT), sigmoidoscopy, and colonoscopy can detect cancer and precancerous polyps, reducing CRC mortality by up to 53% [7, 8]. International guidelines recommend regular screening from ages 50–74 [9] or from age 45 in some regions due to increasing incidence in younger adults [10]. Despite proven benefits, screening rates remain low due to barriers such as limited awareness, fear, and logistical issues [11–14].

Improving uptake depends on a well-structured organizational framework that includes centralized coordination, systematic invitations, electronic health record integration, and clear referral pathways to achieve consistent and equitable program delivery [15, 16]. Effective, multimodal communication strategies are equally important for raising awareness, countering misconceptions, and encouraging action to screening programs [17, 18].

### Economic burden of the CRC

There are several effective screening options available, and the implementation of well-organized screening programs could have a significant impact on reducing the future burden of the disease [19]. The total economic burden of CRC is approximately €19 billion per year for Europe alone and is expected to increase significantly in the coming years [20].

In a recently published guide from the WHO (World Health Organization) Europe, screening for CRC is highlighted as one of the most effective public health interventions, often referred to as the “best investment” in cancer prevention strategies. The guidance encourages countries to set up organized screening programs capable of reaching at least 70% of the eligible population, in order to maximise the benefits of such initiatives [21]. The screening program in the Netherlands had a participation rate of 22% [22]. In a similar initiative conducted in Paris, the FIT yielded a positivity rate of 4.3% among those screened [23].

### Cost-effectiveness from other countries

In Flanders (Belgium), a biennial immunochemical FOBT (iFOBT) screening program for individuals aged 56–74 years was shown to be cost-effective over a 20-year time horizon using a Markov model. The program yielded an incremental cost-effectiveness ratio (ICER) of €1,681 per quality-adjusted life year (QALY) gained for males (95% CI: –€1,317 to €6,601) and €4,484/QALY for females (95% CI: –€3,254 to €18,163), well below the accepted threshold of €35,000/QALY [24]. In Portugal, a cost-utility analysis (CUA) from a societal perspective compared biennial FIT and decennial colonoscopy with no screening. For the target population aged 56–74 years, FIT was cost-effective, with an ICER of €2,694/QALY, whereas colonoscopy was not, with an ICER of €103,633/QALY [25]. Similarly, a French cost-effectiveness analysis using a Markov model compared multiple screening strategies using colonoscopy, flexible sigmoidoscopy, second-generation colon capsule endoscopy, FIT and guaiac-based fecal occult blood test – and revealed that annual FIT screening was the most cost-effective strategy, with an ICER of €48,165 per life-year gained compared with biennial FIT [26].

The cited cost-effectiveness studies of CRC screening, including those conducted in Flanders and other European contexts, were limited by relatively short time horizons (typically 20 years), did not explicitly model the organizational framework of screening delivery, and did not consider the relationship between program costs and the national economic context, such as GDP. Building on this evidence, the present study aims to evaluate the cost-effectiveness of Bulgaria’s national pilot CRC screening program, using a Markov model-based cost-utility analysis with a lifetime horizon of 50 years. It assesses whether early detection through organized, population-based screening offers economic and health benefits compared with no screening, from the payer’s perspective. Additionally, the study explores how organizational and communication strategies influence participation rates and contribute to the program’s overall efficiency and potential for long-term sustainability.

## Methods

### CRC screening campaign – target population, organization model, communication strategy

The 2024 national pilot CRC screening campaign in Bulgaria was a cross-sectional, population-based initiative targeting individuals aged 50–74 years, in line with international guidelines. Eligibility was extended to adults aged 18 + with high-risk profiles, such as a family history of CRC or prior oncological conditions. Free FIT (iFOBT) test kits were distributed through clinical laboratories, national chain of partner pharmacies, general practitioners (GPs), the Regional Health Inspectorate (RHI), and partner organizations. Kits included instructions, a sample container, and a registration form. Participants returned stool samples (with or without the kit) to certified laboratories for qualitative FIT analysis following standardized protocols.

During registration, patients provided information, including age, sex, place of residence, family history, and history of malignancy, to the laboratories. The laboratories anonymized the patients’ data and sent it to the analysing team in compliance with data protection regulations. Patients with negative results were advised to undergo routine rescreening, while those with positive results were referred for a colonoscopy. No colonoscopy outcome data were collected in this program.

**Fig. 1:**
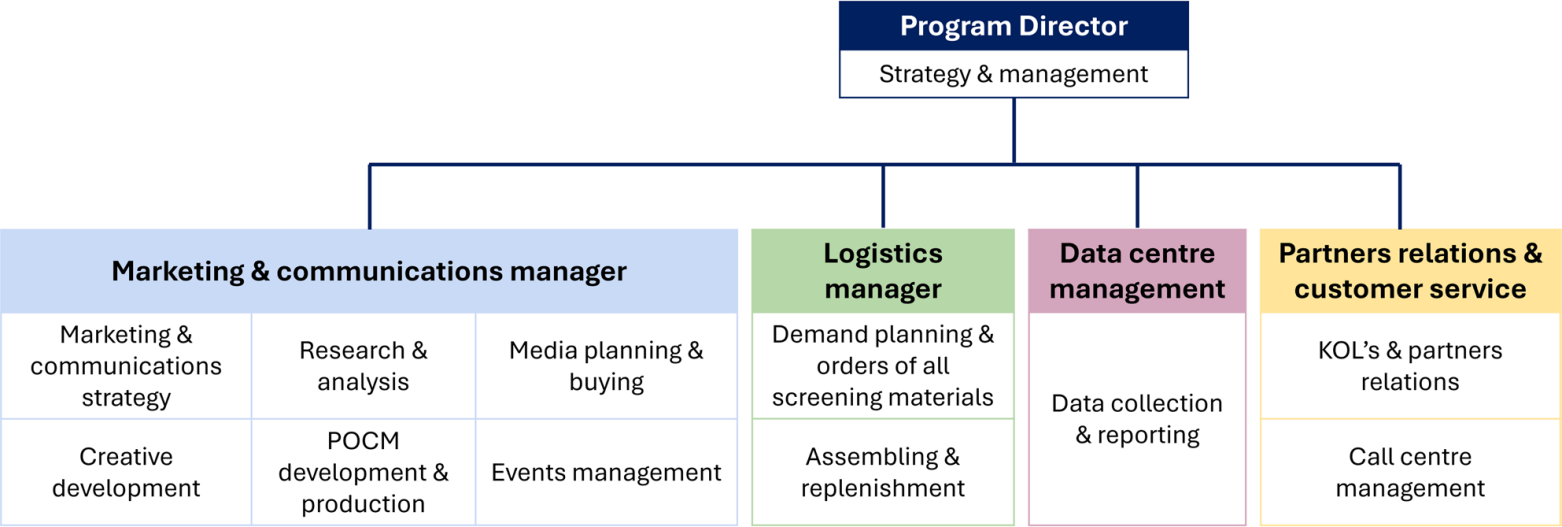
Organization and resources of the colorectal cancer screening program in Bulgaria, 2024. The diagram illustrates the centralized coordination model used in the CRC screening campaign in Bulgaria. The program director oversaw overall strategy and management, supported by four functional leaders: the marketing and communication manager (responsible for campaign strategy, media planning, and public engagement); the logistics manager (in charge of screening kit supply, assembly, and distribution); the data centre manager (focused on data collection and reporting), and the partner relations and customer service manager (managing stakeholder engagement and call centre operations). This structure ensured operational efficiency, cross-functional coordination, and responsiveness throughout the campaign. Abbreviations: CRC – colorectal cancer, KOL – key opinion leaders, POCM – point-of-communication materials

The national CRC screening program was organized through a centrally coordinated model to ensure consistency, efficiency, and a broad population reach by the Lachezar Tsotsorkov Foundation. The organisational model of the screening campaign is presented in Fig. 1. The program director was supported by a team of project managers and domain experts overseeing key operational areas such as logistics, laboratory coordination, data management, and communications. Planning began with a review of international guidelines and consultations with relevant stakeholders, including the formation of an expert advisory board consisting of specialists in gastroenterology and oncology. The program partnered with external professionals to manage various aspects.

**Fig. 2:**
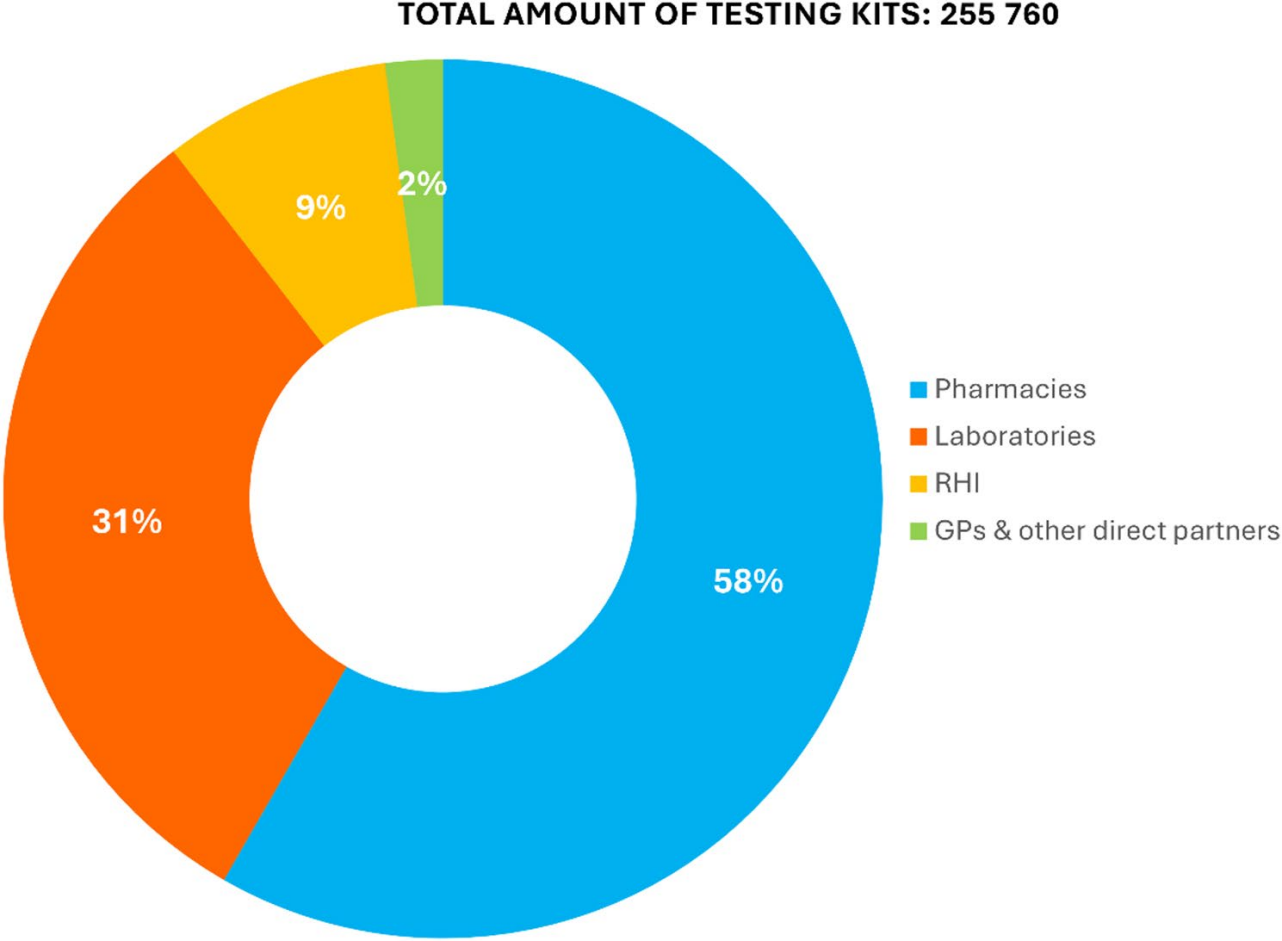
Distribution of screening test kits by distributor in Bulgaria between March and June 2024. Abbreviations: RHI – Regional Health Inspectorate, GP – general practitioner

The information campaign was developed in collaboration with external public relations (PRs), creative and media agencies, selected through a competitive process, and aligned with public health objectives based on market research and stakeholder consultations. A multichannel communication strategy was implemented, combining traditional and digital media with healthcare-based outreach. The key platforms included internet, television, national radio, print media, social media (e.g. Facebook), medical laboratories, and healthcare professionals. The FIT/iFOBT kits used in the pilot screening program were procured by the Foundation and distributed through 321 laboratories, 225 pharmacy locations, additional partner sites, and online channels (Fig. 2). Due to the collaboration with the national pharmacies chain, the kits were positioned according to the best practices of successful merchandising. Various points-of-communication-materials (POCMs) at pharmacies’ hot spots had been used to maximize visibility and off-take, including specially designed displays and directional signs, as well as communication in the monthly magazine distributed free of charge by the chain. Kits were also distributed with every online order during the campaign period.

The program operated on a total budget of €731,340.27 (BGN 1,430,376.91), covering test procurement, distribution, laboratory analysis, logistics, coordination, call center services, mass communication services, media production, media buying, and project management. Budget allocation included 46% for logistics and test materials, 34% for public communications, 5% for professional outreach, and 15% for administrative costs. A flexible, data-driven approach allowed for budget reallocation—shifting funds from media buying to test procurement in response to increased public demand following media exposure.

### Cost-utility analysis – model, input probabilities, heath utilities, costs, sensitivity analysis

**Fig. 3:**
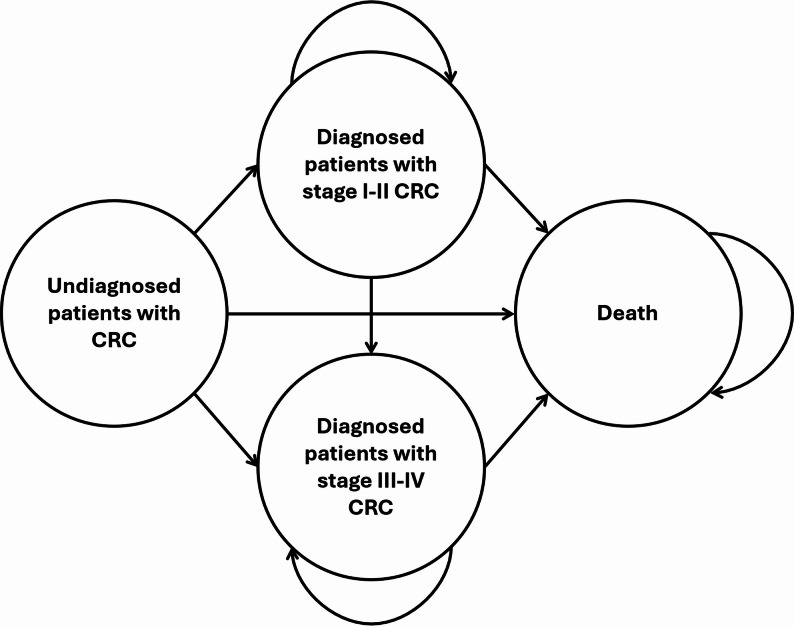
Structure of the Markov model describing health states for colorectal cancer screening outcomes. Abbreviations: CRC – colorectal cancer

To evaluate the cost-effectiveness of the CRC screening program compared with no program, a Markov model was constructed using TreeAge Pro 2023 software. This modelling approach was chosen because it enables the simulation of disease progression through defined health states over time, making it well suited for chronic, multistate conditions such as colorectal cancer, where transitions between health states occur with specific probabilities. Markov models are also widely applied in cost-utility analyses of CRC screening programs [24, 26], supporting the comparability and validity of the present study. The structure of the model is summarized in Fig. 3, which includes the transitions between health states.

The analysis was conducted over a lifetime horizon (50 years) to comprehensively capture the life span of the modelled patients. Each model cycle represents one year, and an annual discount rate of 3.5% was applied to both costs and utilities after the first year. The analysis was conducted from the perspectives of two payers: the Lachezar Tsotsorkov Foundation, which financed and organized the screening program, including the cost for testing, logistics, management and the public awareness campaign, and the National Health Insurance Fund (NHIF), which covered all subsequent healthcare expenditures following a positive test result, including diagnostic procedures, treatment, and follow-up care. Only CRC patients were modelled. The model assumed that all CRC patients, whether screened or unscreened, were eventually diagnosed.

The patients entered the model as undiagnosed patients with CRC. Two arms were analysed: the assessed arm “With screening” and the comparator arm “Without screening”.

The pilot CRC screening program in Bulgaria did not collect data about patients’ CRC status after the screening test. Consequently, the probabilities for transitioning to “diagnosed patient with stage I–II CRC” or “diagnosed patient with stage III–IV CRC” state in the CUA were derived from published data from the Netherlands, SEER, and Denmark, which were considered applicable given the use of comparable screening tests and diagnostic standards within European healthcare systems (Table 1). 

**Table 1:** Literature-based input data for probabilities of stage I–II and stage III–IV colorectal cancer

Strategy/Stage	Stage I-II CRC	Stage III-IV CRC	Reference
Detected within the screening program	67.00%	33.00%	Screening program Netherlands [22]
Detected outside the screening program	46.00%	54.00%	Screening program Netherlands [22]

*Abbreviations*: *CRC* Colorectal cancer

The model also accounted for the annual probability of disease progression from stage I-II to stage III-IV (the natural course of the disease). A Danish study reported 5-year cumulative incidence functions of recurrence for stage I and stage II CRC:

- stage I CRC: 6.80% for colon cancer patients and 9.50% for rectal cancer patients (mean: 8.15%);
- stage II CRC: 11.60% for colon cancer patients and 18.40% for rectal cancer patients (mean: 15.00%).

For cancers detected by screening, recurrence rates were even lower [27]. Based on this data, a mean 5-year cumulative incidence function (CIF) of recurrence for stage I-II CRC as 11.58% (or 2.43% annual incidence after conversion) was calculated.

The model defined “death” as an absorbing health state (patients cannot transition to another state beyond death). No costs or health utilities were accumulated in this state. Stage-specific CRC mortality probabilities were incorporated into the model. According to the SEER data, the 5-year survival rates for patients with stage I-II CRC and stage III-IV CRC were 91% and 45%, respectively (which corresponds to 5-year mortality probabilities of 9% for stage I-II CRC and 55% for stage III-IV CRC). Because the model uses annual probabilities, the 5-year survival rates were converted to annual mortality probabilities using the formula shown in Eq. 1.

Eq. 1. Formula for converting the 5-year survival probability to an annual mortality probability.

$$\begin{aligned} &annual\ mortality\ probability \\&= \mathit{1} - (\mathit{5}\mathrm{-}year\ survival\ probability)^{\wedge}(\mathit{1/5}) \end{aligned}$$

Based on this conversion, the following annual mortality probabilities were calculated:

- stage I-II: 1.87%;
- stage III-IV: 14.76% [28].

The health utilities incorporated in the model were derived from patient health states reported in the literature [29]. Although these data are relatively old, they remain among the most frequently cited sources in cost-utility analyses of colorectal cancer. Their use was considered appropriate because the study population was predominantly White (90%), making it more comparable to the Bulgarian population than more recent studies based mainly on Asian cohorts. Separate average utility values were applied for two disease stages: one for diagnosed patients with stage I–II colorectal cancer and another for patients with stage III–IV disease (Table 2).

**Table 2:** Mean health utilities by stage of colorectal cancer

Stage	Health utilities by stage (QALY)	Mean health utilities by stage (QALY)	Reference
Stage I	0.74	0.74	Ness et al. 1999 [29]
Stage II	0.74		
Stage III	0.67	0.46	
Stage IV	0.25		

*Abbreviations*:* QALY* Quality-adjusted life year

Patients enter the model in the state “undiagnosed patient with CRC”. Depending on the evaluated arm, the modelled patients either undergo screening or do not. A one-time cost of €7.84 (BGN 15.34) per patient is incurred in the “With screening” arm. Patients in the “Without screening” arm do not incur screening costs.

All patients diagnosed with CRC incur colonoscopy costs, estimated from the NHIF perspective at €498.08 [BGN 974.16]. This amount reflects the cost of colonoscopy procedures performed with or without polypectomy or surgical resection in early-stage (stage I–II) disease. At these stages, treatment typically involves endoscopic polypectomy (for malignant polyps) or limited surgical resection, with little or no adjuvant therapy, which is consistent with the National Comprehensive Cancer Network (NCCN) and European Society for Medical Oncology (ESMO) clinical guidelines for colon cancer [30, 31]. The Bulgarian Pharmacotherapeutic Guideline (PTG) for Medical Oncology [32] follows these international recommendations, stating that pharmacotherapy is not required for patients with stage I colorectal cancer and is only occasionally indicated for those with stage II disease. In line with these guidelines, pharmacotherapy costs for early-stage (stage I–II) CRC patients were not included in the analysis.

Pharmacotherapy costs for patients with stage III-IV CRC were based on NHIF data for drug sales. The estimated mean pharmacotherapy costs (6-month treatment) for treatment is €2,031.53 (BGN 3,979.19) per patient for medicines and €4,383.33 (BGN 8,573.04) for treatment administration costs. The total costs of a patient with stage III-IV CRC amounts to €6,417.85 (BGN 12,552.23; including pharmacotherapy and administration).

The model also reports palliative care costs of €1,076.78 (BGN 2,106.00) for patients transitioning to the “death” state.

A probabilistic sensitivity analysis (PSA) was performed to assess the impact of parameter uncertainty on the model outcomes. The analysis used Monte Carlo simulation with 1,000 iterations. Model parameters were assigned probability distributions according to their nature: costs were assumed to follow a normal distribution with a standard deviation (SD) of 20% of the mean value; utility values followed a gamma distribution with a 20% SD; and transition probabilities followed a beta distribution with a 10% SD. The results of the PSA were summarized via cost-effectiveness scatterplots.

In addition, the model underwent internal validation to verify its structural integrity, logical consistency, and reproducibility of outcomes. The simulated results were reviewed to ensure that the transition probabilities and cost-utility outputs aligned with the model’s design assumptions and epidemiological data.

## Results

### Operational performance of the pilot CRC screening program

**Fig. 4:**
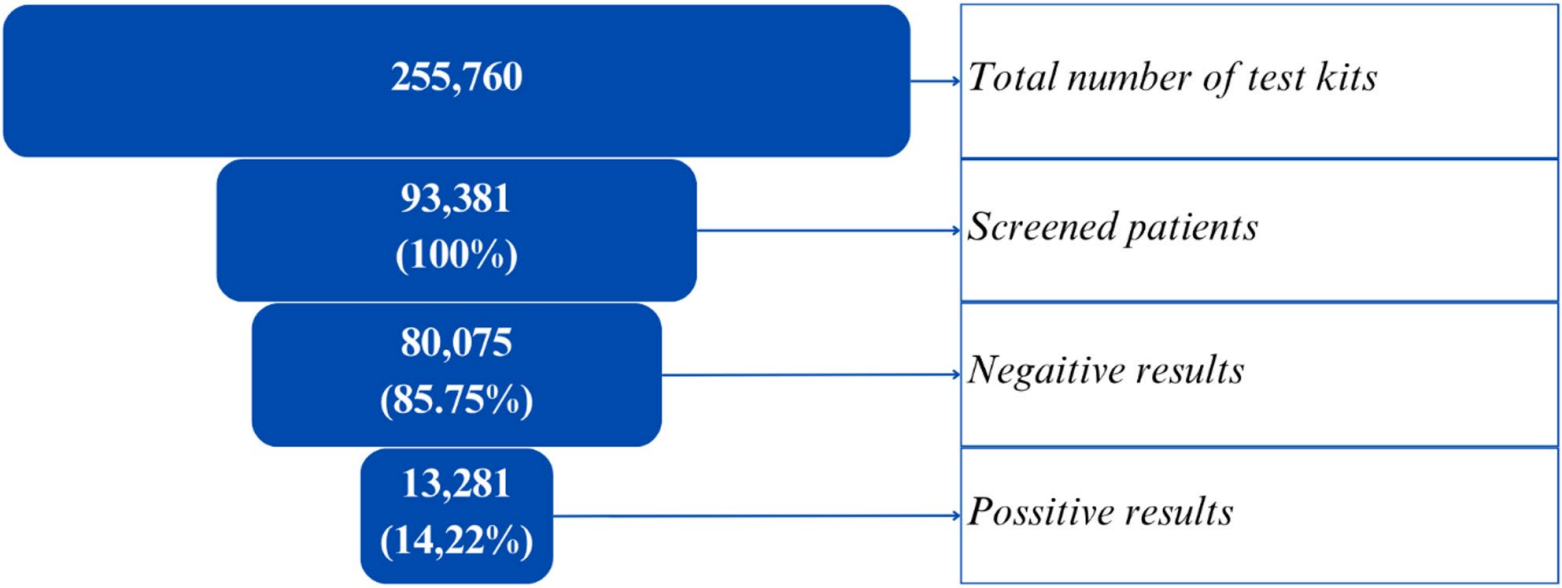
Total number of tests, returned tests, and test results among screened individuals in Bulgaria between March and June 2024

The initial goal of the CRC screening program was to screen 50,000 individuals over a six-month period. By the end of the campaign, 93,381 participants had been screened, surpassing the target by more than 86%. For the purpose of this analysis, the number of tests was considered equivalent to the number of participants, as data on repeat testing were not available. A faeces sample without a kit brought for testing to the laboratory was counted as a free test. Among these, 85.75% yielded negative results, while 14.22% were classified as positive, and ab additional 0.03% (*n* = 25) of the tests produced results deemed suspicious or uncertain, requiring follow-up due to potential technical or biological anomalies (Fig. 4).

Each kit contained instructions, a sample container, and a registration form. The total number of test kits corresponds to the total number of purchased and sent kits. The number of screened patients corresponds to the total number of patients who submitted stool samples in a laboratory, regardless of the use of a test kit (having a test kit is not an obligatory condition for submitting a sample).

The age distribution of the participants in the screening program (Table 3) revealed a mean age of 61.3 years (SD ± 12.4), with sex-specific means of 60.5 years for males and 61.7 years for females, indicating a slightly older female cohort. The sex-based analysis indicated that females exhibited more positive results, although the percentage was lower compared to that of males, with values of 11.9% and 18.2%, respectively (Table 3).

**Table 3:** Number of tests and distribution of positive results by sex and age group among screened individuals in Bulgaria between March and June 2024

	Total		Positive	
	*n*	%	*n*	%
Total	93,381		13,281	
**Sex**				
Female	59,092	63.3%	7,047	53.0%
Male	34,219	36.6%	6,225	46.,9%
Not specified sex	70	0.1%	9	0.1%
**Age**				
18–49 years old	15,121	16.2%	1,280	8.5%
50–60 years old	26,163	28.1%	3,081	11.8%
61–74 years old	39,061	41.9%	6,299	16.1%
75 + years old	12,873	13.8%	2,603	20.2%

Analysis of test outcomes across age groups demonstrated a clear positive link between age and the likelihood of a positive result. Among younger participants aged 18 to 49 years (*n* = 15,131), the positivity rate was 8.5% (*n* = 1,280). The primary target population for CRC screening—individuals aged 50–74 years—accounted for the majority of participants (*n* = 65,224), with a positivity rate of 14.4% (*n* = 9,380), aligning with international benchmarks for average-risk populations. Notably, among participants aged 75 years and older (*n* = 12,873), the positivity rate rose sharply to 20.2% (*n* = 2,603), reflecting the cumulative risk associated with advancing age.

### Communication campaign and communication channels

Analysis of the communication channels revealed that the most influential source of information about the CRC screening campaign was television (TV), which was cited by 45.1% of the participants (*n* = 41,192). The TV campaign was conducted according to the campaign’s purpose and target audience. To achieve the planned coverage, the indicators of the TV campaign were followed on a weekly basis. Based on marketing research, local celebrities (famous actors) were featured in both TV and radio spots, which enforced the effect of the message and encouraged participation in the screening program. Personal networks also played a substantial role, with 24.6% of the respondents (*n* = 22,443) indicating they learned about the campaign through acquaintances or family members. Laboratories served as an informational source for 8.1% of the participants (*n* = 7,417). In addition, general practitioners and other physicians informed 6.6% of the participants (*n* = 5,989) and 14.4% (*n* = 13 143) of the participants were from other sources.

Based on the allocated budget and the number of participants, the cost per participant was calculated at €7.84 (BGN 15.34). This includes not only the cost of the tests but also expenses for procurement, distribution, laboratory analysis, logistics, coordination, call center support, media production and buying, and overall project management.

### Cost-utility analysis and economic impact

The created Markov model quantifies the impact of diagnosing patients at earlier disease stages, showing how this influences associated costs and health utilities. Table 4 presents the cumulative costs and utilities per patient over the full-time horizon.

**Table 4:** Results per patient from the Markov model: discounted final costs and health utilities

Evaluated arm/strategy	Costs, EUR (BGN)	Δ costs, EUR (BGN)	QALY	Δ QALY	ICUR, EUR/QALY
**With screening**	1,137.24 (2,224.24)	-	8.46	-	**The screening program dominates** **(lower costs and more QALYs)**
**Without screening**	1,204.31 (2,355.42)	**−67.07** **(−131.18)**	6.88	**+ 1.58**	

*Abbreviations*: *QALY* Quality-adjusted life year, *ICUR* Incremental cost-utility ratio

Table 5 extends these results to the total estimated number of CRC patients. Since the total number of CRC patients diagnosed through the pilot screening program is not directly available, estimates were derived from the literature, using data from similar programs employing the same type of tests that were used in this program—the FIT tests (data from the Slovenian screening program were used) [31]. This finding indicates that 5.63% of the participants with a positive FIT test result were diagnosed with CRC (862 out of 15,310 people). When this rate was applied to Bulgarian data, where 13,263 participants had positive FIT results, an estimated 747 CRC patients (5.63% of 13,263) were identified. These estimates allow predictions about cumulative utilities for patients diagnosed via the screening program.

**Table 5:** Results for all colorectal cancer patients detected through the screening program: discounted final costs and health utilities

Evaluated arm/strategy	Costs, EUR (BGN)	Δ costs, EUR (BGN)	QALY	Δ QALY	ICUR, EUR/QALY
**With screening**	849,515.95 (1,661,508.39)	-	6,317.43	-	**The screening program dominates** **(lower costs and more QALYs)**
**Without screening**	899,617.56 (1,759,498.61)	**−50,101.61** (-**97**,**990.21)**	5,137.84	**+ 1**,**179.59**	

*Abbreviations*: *QALY* Quality-adjusted life year, *ICUR* Incremental cost-utility ratio

The analysis indicates that the CRC screening program dominates no screening, offering both cost savings (–€67.07 [–BGN 131.18 per patient]) and incremental utilities (+ 1.58 QALY per patient). For the total 747 CRC patients identified through the program, this equates to total cost savings: –€50,101.61 (–BGN 97,990.21); total utilities gained: +1,179.59 QALYs.

**Fig. 5:**
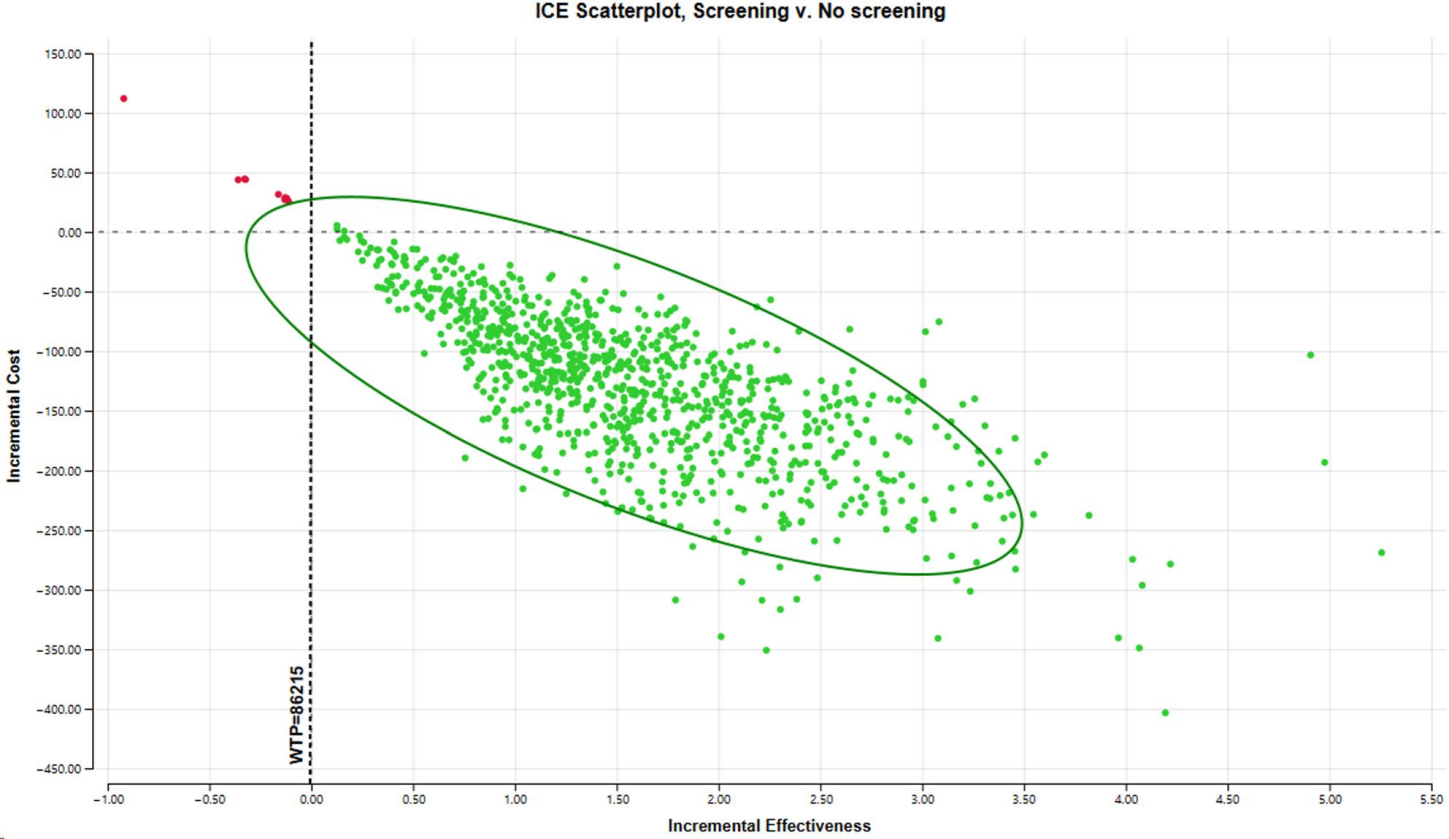
Scatter plot of the probabilistic sensitivity analysis for cost-utility results. Abbreviations: ICE – incremental cost-effectiveness, WTP – willingness-to-pay

The performed PSA confirmed the robustness of the base-case findings. Across 1,000 Monte Carlo iterations, the screening strategy resulted in a mean cost of €1,139.72 (BGN 2,229.10) and a mean effectiveness of 8.51 QALYs, compared with €1,205.68 (BGN 2,358.11) and 6.93 QALYs for no screening. The average incremental result indicated a cost savings of €65.95 (BGN 128.99) and a gain of 1.59 QALYs per individual. The incremental cost-effectiveness (ICE) scatterplot (Fig. 5) demonstrates that most simulations fall in the southeast quadrant, indicating that screening is both more effective and less costly than no screening. At the current willingness-to-pay (WTP) threshold of €44,081.03 (BGN 86,215), corresponding to three times Bulgaria’s GDP per capita, the screening strategy was cost-effective in 99.1% of iterations, confirming the stability of the model results under parameter uncertainty.

The estimated utilities from the Markov model enabled the calculation of QALYs gained for patients of working age. Given that the mean age of the screening participants was 61.3 years and that 56% were of working age (52,321 out of 93,218), it was assumed that 56% of the 747 CRC patients were also of working age—corresponding to 419 individuals. This resulted in a total of + 662.08 QALYs gained for working-age patients (+ 1.58 QALYs per patient). To illustrate the potential economic impact, a theoretical projection was made under the simplifying assumption that one QALY gained corresponds to one full year of productive work capacity. Based on this assumption, the incremental GDP contribution was estimated by multiplying 662.08 QALYs by the GDP per capita of the working-age population (€24,087.00/BGN 47,110.07) (Tables 6 and 7). The resulting theoretical incremental GDP contribution of patients who gained QALYs from the screening program is €15,947,405.22 (BGN 31,190,406.30), equivalent to approximately 0.017% of Bulgaria’s 2023 GDP.

**Table 6:** Gross domestic product per capita in Bulgaria, 2023

GDP per capita in Bulgaria (2023) (NSI data)	
GDP, EUR (BGN)	93,946,530,472.80 (183,743,400,000.00)
Bulgarian working-age population, number	3,900,300
GDP per capita (working-age population), EUR (BGN)	24,087.00 (47,110.07)

*Abbreviations*: *NSI* National Statistical Institute, *GDP* Gross domestic product

**Table 7:** Theoretical gross domestic product contribution based on quality-adjusted life years gained and GDP per capita in Bulgaria

Total QALYs gained for all CRC patients	+ 1,179.59
Percentage of working-age participants, %	56%
QALYs gained for working-age patients	+ 662.08
GDP per capita (working-age population) for 2023, EUR (BGN)	24,087.00 (47,110.07)
Total incremental GDP contribution, EUR (BGN)	15,947,405.22 (31,190,406.30)

*Abbreviations*: *QALY* Quality-adjusted life year, *GDP* Gross domestic product, *CRC* Colorectal cancer

In order to calculate the potential savings from treating patients in advanced stages of CRC due to the successful introduction of a screening program, data from the Netherlands are used to distribute newly diagnosed patients by stage depending on whether cases are detected within a screening program or not. In a world without a screening program, 54% of new patients are diagnosed with stage III-IV, whereas in a world with a screening program, the percentage of these patients is 33% (Table 1). Using these data, it is possible to establish the avoided number of cases of CRC diagnosed with stage III-IV and the saved treatment costs at this advanced stage (the cost of treatment per patient in stage III-IV was calculated as €6,417.85/BGN 12,552.23). For the calculation of the population in the future period, an annual decrease in the population in Bulgaria of −1.147% was used, which was based on data from the National Statistical Institute (NSI). The incidence of CRC is, on average, 0.431/1,000 for men and women and is based on data from a previous study, which also contains data for Bulgaria [33]. The expected target group for screening is taken into account: the population of Bulgaria between 50 and 74 years of age. A total of €6,341,213.74 (BGN 12,402,336.06) is calculated. Savings as a result of avoided cases of advanced CRC due to timely diagnosis in the period 2024–2028 (Table 8).

**Table 8:** Estimated cost savings from avoided cases of newly diagnosed advanced colorectal cancer, 2024–2028

Year	2024	2025	2026	2027	2028	Total 2024–2028
Population 50–74 years, number	2 233 977	2 208 353	2 183 023	2 157 984	2 133 232	-
Morbidity (new cases)	963	952	941	930	919	4 705
Morbidity (new cases stage III-IV) without screening	520	514	508	502	496	2 541
Morbidity (new cases stage III-IV) with screening	318	314	310	307	303	1 553
Number of avoided cases of stage III-IV CRC with screening	202	200	198	195	193	988
Saved expenditure from avoided cases of CRC in persons 50–74 years old, EUR (BGN)	1,297,671,85 (2,538,025.53)	1,282,390.15 (2,508,914.38)	1,268,073.98 (2,480,137.13)	1,253,529.17 (2,451,689.96)	1,239,151.19 (2,423,569.07)	6,341,213.73 (12,402,336.06)

## Discussion

The 2024 pilot CRC screening program in Bulgaria provided critical insight into the feasibility, efficiency, and impact of organized population-level screening for a high-burden malignancy. The findings underscore the importance of structured planning, multichannel outreach, risk-adapted inclusion criteria, and robust logistical coordination as essential elements for the success of public health interventions [15, 16].

First, centralized coordination proved critical. Coordination by the Lachezar Tsotsorkov Foundation allowed rapid mobilization of resources and ensured standardized procedures across all implementation points. Central oversight of logistics, laboratory analysis, and data collection minimized variability, guaranteed quality control, and enabled real-time adaptation to participation trends. This framework demonstrates that large-scale screening can be efficiently organized even outside the formal health system when oversight, accountability, and data transparency are maintained [34].

Second, the campaign’s success was strongly driven by a multichannel communication strategy, with TV serving as the primary driver of participation (45.1%) [35]. TV outreach effectively conveyed key messages on CRC risk and prevention, whereas complementary channels—social networks, personal referrals, and healthcare providers—expanded reach to diverse demographic groups [36]. This layered communication model confirmed that combining mass media with interpersonal influence maximizes engagement and participation, particularly in populations with heterogeneous access to information.

Third, test kits were made widely available through both healthcare and community access points, including partner laboratories and a national pharmacy chain. This approach removed typical logistical barriers, such as travel time and institutional constraints, by enabling home sample collection and centralized laboratory processing. The model proved both convenient for participants and efficient for administrators, and it provides a replicable structure for scaling national CRC screening within existing infrastructures.

Finally, flexible, data-driven budgeting was essential for optimizing program efficiency. The budget in the pilot program was continuously restructured based on participation data, enabling dynamic reallocation of resources between communication, logistics, and test supply. This flexibility ensured optimal use of funds and significantly reduced per-participant costs to €7.84 (15.34 BGN)—ten times lower than the €76.69 (150 BGN) estimated in Bulgaria’s National Cancer Plan. The experience illustrates that real-time monitoring and adaptive budgeting can enhance both efficiency and financial sustainability in public health interventions [37].

Within just three months, nearly 100,000 individuals were screened—almost quadrupling the targets set by the National Cancer Plan. This compares favourably to participation rates in other European programs, including those in Paris, the Netherlands, and Slovenia [22, 23, 38]. The Bulgarian experience shows that high-volume CRC screening is achievable even in resource-limited environments, provided that strategic coordination and communication mechanisms are in place.

The FIT test positivity rate of 14.22%, with men showing significantly higher rates (18.19% vs. 11.93%), supports future tailoring of outreach toward male populations. The program’s clear referral pathway to colonoscopy and adherence to international best practices [10] further strengthen its operational robustness.

Economic analysis confirmed that, compared with the no-screening scenario, the pilot program achieved both cost savings and health gains. The model projected a reduction of €67.07 per patient and an average gain of 1.58 QALYs, which is consistent with cost-effectiveness findings from comparable European analyses [24–26]. The extrapolation of results indicated potential healthcare savings of €33.1 million (BGN 64.8 million) and an incremental GDP contribution of €15.9 million, assuming full productivity recovery.

### Limitations and areas for improvement

Despite the campaign’s successes, several limitations must be acknowledged. The reliance on anonymized data, while essential for privacy protection, prevented individual-level follow-up and verification of colonoscopy outcomes. Consequently, it was not possible to confirm how many FIT-positive participants proceeded to diagnostic colonoscopy or to determine the cancer stage distribution. Future programs should ensure integration with national electronic health records to allow longitudinal tracking and quality control.

Participation patterns also revealed underrepresentation of men despite higher positivity rates, suggesting the need for tailored communication strategies and male-targeted engagement. Additionally, long-term program sustainability will depend on consistent funding, improved data management, and expanded diagnostic capacity. International experience, such as the Slovenian model, demonstrates that the continuity of CRC screening relies on stable institutional frameworks and ongoing monitoring [38].

Several assumptions underpin the cost-utility model and must be interpreted with caution. First, the stage distribution and CRC detection rates were informed by international data, primarily from the Netherlands, SEER, and Slovenia, due to limited local evidence. While these sources reflect similar epidemiological contexts, they may not fully capture Bulgaria’s disease dynamics [39]. Second, early-stage CRC treatment costs excluded drug therapy, which is in line with clinical practice, where pharmacological treatment is rarely indicated. However, this assumption may slightly underestimate total treatment costs. Third, the conversion of QALY gains into GDP contribution assumes a one-to-one correspondence between QALY and full working capacity, which likely overstates productivity effects. The sensitivity analysis confirmed the robustness of these results, but nationally derived data would allow even greater model precision. Future economic evaluations would benefit from using national data sources to estimate key parameters, including probabilities, utility values, and productivity effects.

Beyond methodological considerations, the results of this pilot carry important implications for national policy design and health system planning. Because the program was organized and financed by a private foundation rather than within the public health system, its results cannot be directly generalized to publicly funded national programs. Nevertheless, the demonstrated success of centralized coordination, FIT-based testing, and data-driven communication offers a transferable model for similar European contexts. The findings highlight the feasibility of implementing large-scale, cost-effective CRC screening in low- and middle-resource environments when strong organizational and monitoring mechanisms are in place.

To ensure sustainable national implementation, Bulgaria’s health authorities should (1) integrate screening and follow-up data into national electronic health and cancer registry systems that are open-access and available to all relevant stakeholders; (2) ensure stable public funding to transition from foundation-based to government-supported screening; (3) expand colonoscopy and pathology capacity to meet expected demand; (4) implement targeted communication campaigns, particularly to increase participation among men and lower-income groups; and (5) strengthen the engagement of general practitioners, whose participation in the pilot was limited to only 2% of screened individuals, by introducing incentives and clearer institutional support for their active role in promoting and facilitating screening. Together, these measures would support the transition from foundation-led to system-integrated CRC screening and improve long-term population health outcomes.

## Conclusion

The national pilot CRC screening campaign in Bulgaria has provided powerful evidence that organized, population-based screening is both feasible and highly impactful in the local context. By combining inclusive eligibility criteria, cost-effective logistics, and a robust outreach strategy, the program achieved an almost fourfold increase over national screening targets.

The CRC screening program in Bulgaria demonstrated substantial health and economic benefits. The cost of this pilot screening program is justified, as it results in cost savings and improved health benefits for CRC patients. Early screening facilitates timely diagnosis and treatment of asymptomatic patients, thereby avoiding the more expensive later stages of treatment.

## Data Availability

The data supporting the conclusions of this article are included within the article.

## Abbreviations

CIF: Cumulative incidence functions
CRC: Colorectal cancer
CUA: Cost-utility analysis
ESMO: European Society for Medical Oncology
EU: European Union
FIT: Fecal immunochemical test
FOBT: Fecal occult blood test
GDP: Gross domestic product
GP: General practitioner
ICE: Incremental cost-effectiveness
ICER: Incremental cost-effectiveness ratio
ICUR: Incremental cost-utility ratio
iFOBT: Immunochemical faecal occult blood test
KOL: Key opinion leaders
NCCN: National Comprehensive Cancer Network
NHIF: National Health Insurance Fund
NSI: National Statistical Institute
POCM: Point-of-communication materials
PR: Public relations
PSA: Probabilistic sensitivity analysis
PTG: Pharmacotherapeutic Guideline
QALY: Quality-adjusted life year
RHI: Regional Health Inspectorate
TV: Television
WHO: World Health Organization
WTP: Willingness-to-pay

## Declarations

The organizational and the analytical teams received anonymized data from the laboratories, so no ethics committee approval was required for the analysis.
